# Comparison of ^68^Ga-DOTATATE Positron Emmited Tomography/Computed Tomography and Gadoxetic Acid-Enhanced Magnetic Resonance Imaging for the Detection of Liver Metastases from Well-Differentiated Neuroendocrine Tumors

**DOI:** 10.3390/curroncol31010036

**Published:** 2024-01-16

**Authors:** Moran Drucker Iarovich, Ricarda Hinzpeter, Brian Michael Moloney, Katrina Hueniken, Patrick Veit-Haibach, Claudia Ortega, Ur Metser

**Affiliations:** 1 Joint Department of Medical Imaging, University Health Network, Sinai Health Systems, Women’s College Hospital, University of Toronto, Toronto, ON M5R 0A3, Canada; moran.druckeriarovich@uhn.ca (M.D.I.);; 2 Department of Biostatistics, University Health Network, Toronto, ON M5G 2C4, Canada

**Keywords:** ^68^Ga-DOTATATE, PET, MRI, gadoxetic acid, liver, metastases, neuroendocrine tumors

## Abstract

This study aimed to compare the detection of neuroendocrine tumor liver metastases (NLMs) in hepatobiliary-specific contrast-enhanced MRI (pMR) versus ^68^Ga-DOTATATE PET/CT (DT-PET). This retrospective study cohort included 30 patients with well-differentiated neuroendocrine tumors who underwent both DT-PET and pMR. Two readers independently assessed NLMs count, SUVmax on DT-PET, and signal characteristics on pMR. A consensus review by two additional readers resolved discrepancies between the modalities. Results showed concordance between DT-PET and pMR NLM count in 14/30 patients (47%). pMR identified more NLMs in 12/30 patients (40%), of which 4 patients showed multiple deposits on pMR but only 0–1 lesions on DT-PET. DT-PET detected more in 4/30 patients (13%). Overall, pMR detected more metastases than DT-PET (*p* = 0.01). Excluding the four outliers, there was excellent agreement between the two methods (ICC: 0.945, 95%CI: 0.930, 0.958). Notably, pMR had a higher NLM detection rate than DT-PET, with correlations found between lesion size on pMR and DT-PET detectability, as well as diffusion restriction on pMR and SUVmax on DT-PET. In conclusion, in consecutive patients with well-differentiated NETs, the detection rate of NLM is higher with pMR than with DT-PET. However, when excluding patients whose tumors do not overexpress somatostatin receptors (13% of the cohort), high concordance in the detection of NLM is observed between DT PET and pMR.

## 1. Introduction

Neuroendocrine tumors (NETs) are a heterogeneous group of malignancies arising most commonly from the digestive and respiratory tracts [1,2]. Their incidence has continuously risen over the past decades. These are usually slow-growing tumors, and prognosis depends on several factors, including excess hormone production, tumor grade, Ki-67 or MIB-I proliferation index, initial stage, and surgical resectability [3,4]. Approximately 40–50% of NET patients will have liver metastases upon initial presentation, and up to 75% of NETs will develop liver metastases throughout the natural course of disease progression [5,6,7,8]. Neuroendocrine liver metastases (NLMs) are a poor prognostic marker, especially when not amenable to surgical resection or ablative therapy. Early detection and accurate delineation of metastatic disease are crucial for determining the most appropriate treatment. These include liver-directed therapies such as surgery, percutaneous ablation, or arterial chemoembolization, depending on the extent and distribution of hepatic metastatic disease [1,6,7,9,10].

A consensus is still lacking regarding the best imaging approach for detecting NLMs. Morphological imaging techniques, including CT and MRI, and functional imaging using somatostatin receptor (SSTR)-based imaging are considered valid investigations in current guidelines [11]. In recent years, PET with ^68^Ga-DOTA –peptides such as ^68^Ga-DOTATATE has become the gold standard for the evaluation of SSTR-positive disease in patients with NETs [12,13,14,15,16,17,18]. Magnetic resonance imaging (MRI) is regarded as a superior modality for liver metastases detection, providing excellent anatomic details [19,20]. The use of hepatobiliary-specific contrast agents like gadoxetate disodium (Primovist/Eovist) further improves metastases detection compared to traditional gadolinium-enhanced MRI [20,21,22,23,24]. Primovist targets hepatocytes through organic anion transporter protein 1B3 (OATP1B3) expression [22,25]. It behaves similarly to conventional gadolinium agents during dynamic post-contrast imaging but has an additional hepatobiliary phase (HBP) approximately 20 min after injection. During HBP, normal liver tissue exhibits T1 shortening due to hepatocyte uptake, allowing for clear differentiation from metastases that lack hepatocytes. This enhanced spatial resolution greatly aids in identifying small liver lesions, impacting management in a significant proportion of patients being considered for surgical resection [22,25,26].

Data comparing the diagnostic performance of ^68^Ga-DOTATATE PET/CT to extracellular gadolinium-enhanced MRI for the detection of NLM is limited, with existing studies showing variable results [27,28,29,30], and, to the best of our knowledge, no direct comparison of hepatobiliary-specific contrast-enhanced MRI and ^68^Ga-DOTATATE PET/CT is available. The purpose of this study was to conduct a head-to-head comparison of the detection of NLM with gadoxetate disodium-enhanced MRI versus ^68^Ga-DOTATATE PET/CT.

## 2. Material and Methods

### 2.1. Patient Population and Study Design

This retrospective study was approved by the institutional ethics review board, and informed consent was waived. Consecutive patients who underwent both gadoxetate disodium (Primovist)-enhanced MRI (pMR) and ^68^Ga-DOTATATE PET/CT (DT-PET) within 4 months between January 2018 and December 2021 were included. All patients had histological proof of a well-differentiated (G1–G3) NET. Clinical and histopathological parameters were recorded, including age, gender, primary site of NET, any prior treatment received, tumor grade, and Ki-67 index.

### 2.2. Image Acquisition

#### 2.2.1. Primovist-Enhanced MRI

pMR was performed on 1.5T (Magnetom Avanto; Siemens Healthcare, Erlangen, Germany) or 3T MR system (Magnetom Verio with Tim system; Siemens Health care, Erlangen, Germany) with multichannel phased array coils (16 or 32 channels) using our institutional standard liver p-MR protocol, as previously described [31]. In brief, axial and coronal T2 HASTE, echo-planar imaging two-dimensional diffusion (b 100, 600), precontrast and postcontrast 3D, fat-saturated gradient echo T1-weighted volumetric interpolated breath-hold examinations (VIBE) before contrast, and after intravenous injection of 0.025 mmol/kg gadoxetic acid in the arterial phase, portal venous phase, transitional phase, and the hepatobiliary phase (20 min post-contrast injection).

#### 2.2.2. ^68^Ga-DOTATATE Positron Emission Tomography/Computed Tomography

PET was performed on a Biograph mCT 40 scanner (siemens Healthcare, Erlangen, Germany). Patients were positioned supine with their arms outside of the region of interest. Images were obtained from the top of the skull to the upper thighs 64 ± 16.5 min (mean ± SD) after injection of 135 ± 25 (mean ± SD) MBq of ^68^Ga-DOTATATE (Canprobe; Toronto, ON, Canada). During uptake time, water-soluble oral contrast was given for bowel opacification on CT. Low-dose CT without intravenous contrast was used for attenuation correction as per standard departmental protocol. CT parameters were 120 kV; 3.0 mm slice width, 2.0 mm collimation; spiral pitch factor, 1.2. PET emission scan using time of flight with scatter correction was obtained, covering the identical transverse field of view. Image size: 2.6 pix size; slice 3.27; 5mm FWHM Gaussian Filter type. Overall, 5–9 bed positions were obtained as per patient height (2–5 min/bed position).

### 2.3. Data Abstraction

For each subject, pMR and DT-PET were assessed independently by 2 experienced fellowship-trained observers. Readers were blinded to all clinical information. The first reader assessed the pMR scans and was blinded to DT-PET results, while the second reader analyzed DT-PET studies and was blinded to pMR results. The readers were presented with all available sequences of their assigned cases to allow differentiation of potential metastatic lesions from incidental benign entities. The number of metastases per segment (0–4 or ≥5) was recorded. On pMR, for the dominant lesion in each segment, size, T2 signal intensity, presence of arterial enhancement, contrast washout, hypointensity in the hepatobiliary phase, and the presence of diffusion restriction were recorded. Readers were instructed to register only lesions clearly visible/identifiable per each sequence. On DT-PET, SUVmax and the modified Krenning score of the dominant metastasis in each segment were documented. The Krenning score is a semi-quantitative method originally used to assess the degree of tracer uptake on octreotide scintigraphy. The score compares tumoral tracer uptake to the uptake in the liver and spleen. The score ranges from 0 to 4, with higher scores indicating greater uptake. In recent years, the use of the Krenning score has become more widespread, with the advancements and growing use of other somatostatin receptor imaging techniques, including PET-CT with DOTA-peptides (referred to as modified Krenning score). If more than one metastasis per segment was encountered, minimal SUVmax and the lowest modified Krenning score were also recorded. For both the pMR and DT-PET datasets, a second review was performed in consensus by a panel of two additional readers (UM and MDI with 20 years and 7 years of experience, respectively), settling discrepancies in the number and location of metastases on each modality. An agreement between all readers regarding potential liver metastasis had to be achieved in order for the lesion to be recorded for research analysis. Any questionable lesion or any difficulty in lesion identification would exclude the lesion from being registered as hepatic metastasis.

### 2.4. Reference Standard

The final imaging datasets were compared to a composite reference standard (best available, by the following scale): histopathology including from surgical specimen or biopsy in 18/30 (60%) patients; follow-up imaging in 29/30 (97%) patients and/or concordance between DT-PET and pMR on a segmental level in 14/30 (47%) patients. In one patient who had no metastases on DT-PET and pMR, the reference standard was concordant between the two exams only.

### 2.5. Statistical Analysis

The numbers of metastases detected on each test (pMR and DT-PET) were tabulated. To measure agreement between tests, a weighted Cohen’s Kappa was computed for ordinal data at the segment level. The data was treated ordinally to account for censored counts (5 or more metastases in each segment) where exact numbers were not measured. A multilevel, multirater Kappa coefficient was computed [32] for repeated-measures data, as each patient’s metastasis counts were measured in 8 segments. Kappa statistics were benchmarked using the following rule: <0 indicated no agreement; 0 to 0.2 indicated slight agreement; 0.21 to 0.4 indicated fair agreement; 0.41 to 0.6 indicated moderate agreement; 0.61 to 0.8 indicated substantial agreement; and 0.81 to 1.0 indicated near-perfect agreement [33].

A Wilcoxon signed-rank test for paired data was performed at the patient level to identify differences in the number of metastases detected on pMR versus DT-PET. To compute the total number of metastases detected per patient, segments with 5 or more metastases were assigned a value of 5, and counts were summed across all segments. A sensitivity analysis was conducted, removing major outliers, as these cases were deemed to be clinically dissimilar from most studied cases. Proportions of patients with greater, equal, or fewer numbers of metastases on DT-PET versus pMR were then described, with and without these outliers. Among segments with metastases detected on both tests, unadjusted linear mixed-effects regression models were fit to examine associations between SUV measures and pMR features. SUV values were log-transformed; effect estimates describe the percent change in SUV associated with each pMR feature. Unadjusted logistic regression with mixed effects was then used to examine associations between pMR features and detection of any metastases on DT-PET (binary variable, ≥1 versus 0 detected metastases). In all mixed-effects models, a random intercept was included for each patient. All statistical tests were two-sided, using alpha < 0.05 to define statistical significance. Analyses were conducted using R statistical software version 4.1.2.

## 3. Results

**Figure 1:**
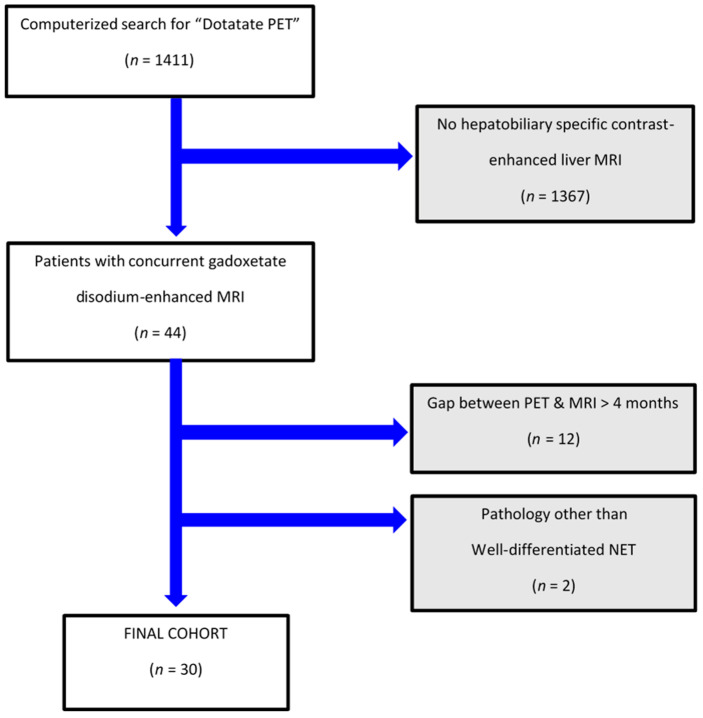
Patient flowchart.

There were 1411 patients who underwent DT-PET during the study interval, of which 44 individuals also had pMR. Fourteen of them were excluded as they did not fit the inclusion criteria (see patient flowchart; Figure 1). Overall, our study population included 30 patients, including 18 women (60%) and 12 men (40%) with a mean age (±SD) of 57 (±12) years with a median duration of clinical and imaging follow-up for NET of 4.8 years (IQR 3.6, 7.8). Specifically, the study cohort had imaging and clinical follow-up for NET beyond study-referenced scans of a median of 3 years (IQR 2.4, 3.8).

**Figure 2:**
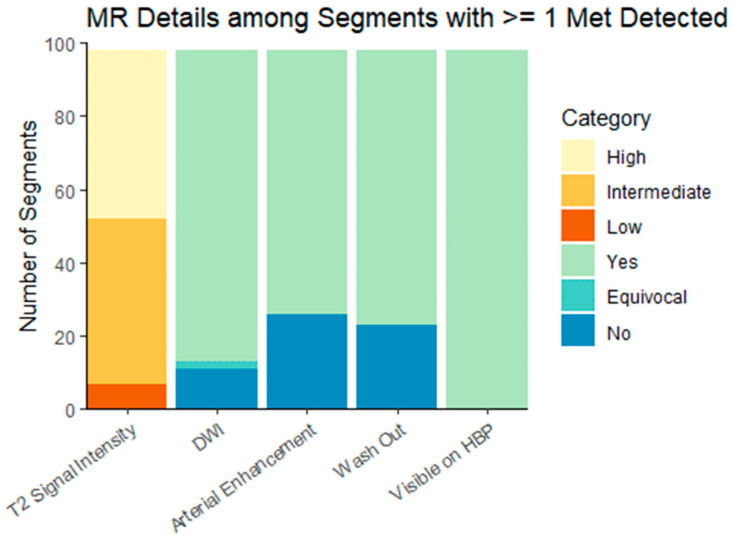
Hepatobiliary-specific contrast-enhanced MR imaging characteristics of liver metastases. DWI = diffusion-weighted imaging; HPB = hepatobiliary phase. Characteristics are presented for the dominant lesion in each liver segment with at least one metastasis.

pMR preceded DT-PET in 18 patients and followed DT-PET in 12, with a mean (±SD) time interval of 13 (±54) days between the tests. Cohort characteristics are summarized in Table 1. There were 9/30 (30%) patients referred for initial staging of a newly diagnosed NET and 21/30 (70%) referred for imaging following prior surgical intervention or systemic therapy. There were 14/30 (47%) patients with metastatic disease limited to the liver and 9 /30 (30%) patients with hepatic and extrahepatic metastases. No metastases were detected in the remaining 7/30 (23.3%) patients on any imaging modality. According to the classification of disease extent in the ENETS guidelines [9], 11/30 (37%) patients had diffuse pattern metastatic, 9/30 (30%) had simple pattern NLMs, and 3/30 (10%) had complex pattern NLMs. The size of metastases on pMR was 1.5 ± 1.2 cm (range: 0.4, 7.4). The imaging characteristics of metastases on DT-PET and pMR are summarized in Table 2 and Table 3 and Figure 2.

**Figure 3:**
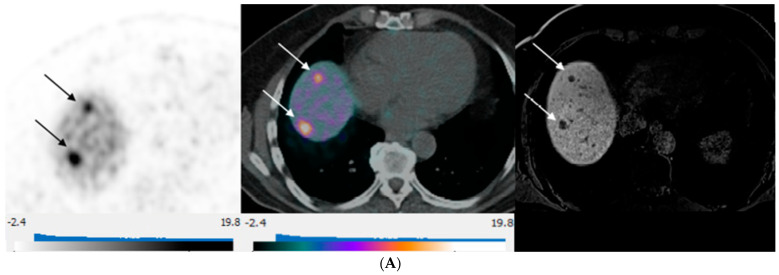

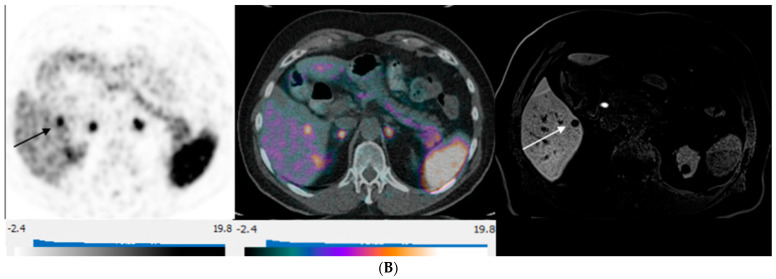
80-year-old man with well-differentiated G2 small bowel neuroendocrine tumor referred for restaging. ^68^Ga-DOTATATE PET/CT and Hepatobiliary-specific contrast-enhanced MR show a similar number and distribution of metastases. (**A**) Axial ^68^Ga-DOTATATE PET (**left**), fused PET/CT (**middle**), and corresponding hepatobiliary phase T1 VIBE image (**right**) show intense focal radiotracer uptake (arrows) in two small segment 8 metastases. (**B**) Axial ^68^Ga-DOTATATE PET (**left**), fused PET/CT (**middle**), and corresponding hepatobiliary phase T1 VIBE image (**right**) show intense focal radiotracer uptake in a small segment 5 deposit (arrows).

**Figure 4:**
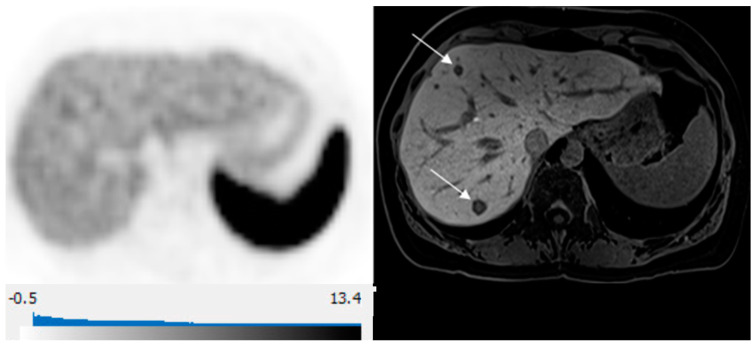
65-year-old woman with well-differentiated G2 lung NET, 3 years following primary surgery. Axial ^68^Ga-DOTATATE PET at the liver dome (**left**) shows no focal radiotracer uptake corresponding to the 2 metastases depicted on corresponding axial hepatobiliary phase gadoxetic acid-enhanced T1 VIBE image (**right**; arrows) to suggest liver metastases over-expressing somatostatin receptors type 2.

Equal NLM counts between DT-PET and pMR were observed in 14 patients (14/30, 47%) (Figure 3). A greater number of deposits were identified on pMR in 12/30 patients (40%), and a greater number of metastases were detected on DT-PET in 4/30 (13%) of patients. Overall, for the entire patient population, pMR detected more metastases than DT-PET (*p* = 0.01). Of the 12 patients with a greater count of liver metastases on pMR, there were 4 patients in whom multiple deposits were seen on pMR but only 0–1 lesions on DT-PET (Table 4 and Figure 4). All four patients had tumors that were grade 2 or 3 with a Ki 67 index of 5–30%. When these four patients were excluded, no significant differences were found between the number of lesions detected on pMR versus DT-PET (*p* = 0.10).

**Figure 5:**
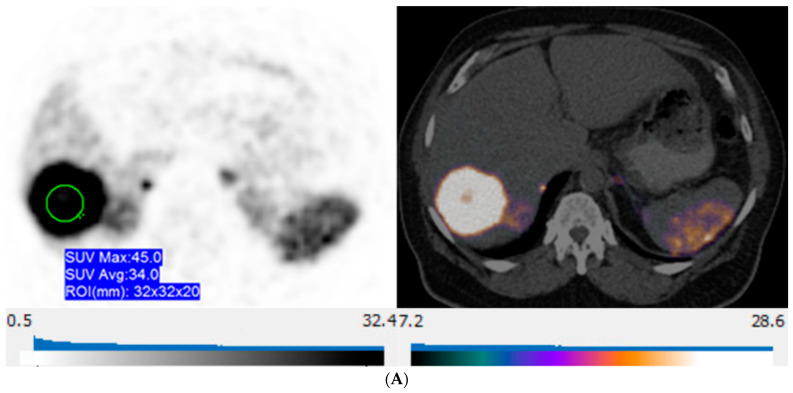

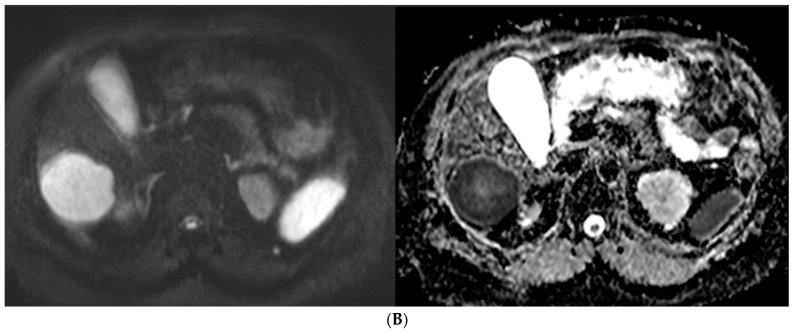
57-year-old man with well-differentiated G2 pancreatic neuroendocrine tumor referred for initial staging. (ADC = Apparent diffusion coefficient). (**A**) Axial ^68^Ga-DOTATATE PET (**left**) and fused PET/CT (**right**) images show intensely ^68^Ga-DOTATATE avid segment 6 deposit (green circle within metastasis represents region of interest, SUVmax, 45.0). (**B**) High signal intensity is seen on diffusion-weighted MR image with b value = 600 s/mm^2^ (**left**), and corresponding image on ADC map shows marked hypointensity (**right**).

Overall, DT-PT and pMR showed substantial agreement (Cohen’s kappa score: 0.757 (0.538, 0.975)). When the four outliers were excluded, agreement between the studies was nearly perfect (Kappa score: 0.945 (0.910, 0.980)). A correlation was found between lesion size on pMR and detection on DT-PET (odds ratio = 3.28; 95%CI: 1.1, 9.84). No significant relationships between modified Krenning score on DT-PET and T2 signal intensity, DWI, arterial enhancement, or washout were seen. Metastases with restricted diffusion on pMR had higher maximal SUVmax on DT-PET (*p* = 0.003) (Figure 5).

## 4. Discussion

DT PET and pMR are sensitive imaging modalities for the identification of NLM. In a heterogeneous patient population with variable tumor grade and Ki-67 index, there was substantial agreement between the modalities (Kappa score 0.757), with a higher sensitivity for detecting NLM on pMR. When excluding four outliers who had grade 2 or 3 tumors and showed no or low ^68^Ga-DOTATATE avidity on PET, there was a very strong agreement between pMR and DT-PET in the detection of NLM (Kappa score, 0.945).

The presence of liver metastases is an important prognostic factor in patients with NET, regardless of the primary site of disease [34]. The management of NETs metastatic to the liver often requires a multidisciplinary approach. Mapping of disease extent in the liver is crucial for appropriate therapy selection and therapy planning. When feasible, surgical resection of NLM offers the best long-term outcomes. This can be offered to patients with good performance status, grade 1 or 2 tumors, without extrahepatic disease, and where resection of >90% of metastatic tumor burden is feasible with adequate volume of a future liver remnant. The use of tumor ablation may expand the number of patients suitable for surgical debulking. For those who are not candidates for surgery, liver-directed therapies, including transarterial chemoembolization and radioembolization, may provide local disease control and symptomatic relief [10].

Imaging, especially liver MRI, plays a crucial role in delineating disease extent, determining resectability, and informing liver-directed therapies. Prior studies comparing the performance of DT-PET to conventional imaging in the staging of patients with NETs have reported superior diagnostic performance of DT-PET over CT [16,35]. There are only limited studies comparing DT-PET and MRI in the detection of NLM with varying results. Jackson et al. evaluated 32 patients with DT-PET and conventional imaging (CT and/or MRI) and found that three-quarters of patients’ DT-PET detected more disease in the liver than CT or MRI, although only half of the patients in that study had MRI [27]. In contradiction, Haider et al., who assessed 32 patients with DT-PET and MRI for liver metastases with an emphasis on diffusion-weighted (DWI) and gadoteridol-enhanced dynamic contrast-enhanced (DCE) sequences, showed increased detection rates with MRI (28% and 33% for DWI and DCE, respectively) [28]. Furthermore, a recent meta-analysis comparing the diagnostic performance of ^68^Ga-DOTA-SSA PET/MRI to ^68^Ga-DOTA-SSA PET/CT in detecting liver metastases in patients with NETs reported a higher pooled detection rate of 93.5% (95% CI, 85.1–97.3%) and 76.8% (95% CI, 64.8–85.6%) for PET/MR and PET/CT respectively, indicating MR significantly contributes to the detection of NLM [36]. This meta-analysis included six studies with 8–30 patients in each study and a total of 638 liver metastases [37,38,39,40,41,42]. Tumor grade and Ki 67 index were not documented.

The combination of diffusion-weighted imaging and hepatobiliary phase has been shown to yield the highest sensitivity and specificity in the detection of NLM, suggesting MR with hepatobiliary contrast agents is the modality of choice for assessment of disease extent in the liver in patients with NETs [43]. Our study confirms these findings with the highest detection of NLM on the hepatobiliary phase and DWI (Figure 4). A direct comparison of hepatobiliary-specific contrast-enhanced MR and conventional extracellular gadolinium-enhanced MRI has shown a significant impact of MR with hepatobiliary-specific contrast on the surgical management of patients with liver metastases [44]. To our knowledge, no prior studies compared hepatobiliary-specific contrast-enhanced MR and DT-PET in the detection of NLM. Our study demonstrates a similar diagnostic strength for both modalities in detecting NLM for tumors overexpressing somatostatin receptors but highlights the limitation of DT-PET when the somatostatin-receptor expression is low, and in these cases, pMR is crucial in accurately delineating disease extent. This is in line with prior reports of inverse correlation between tumor Ki67 index and SUVmax on ^68^Ga-DOTA-SSA PET [45]. Further studies comparing the utility of these modalities in NLM, especially for patients with intermediate or high Ki-67 index, are needed. We propose that future trials should assess the use of combined ^68^Ga-DOTATATE PET/gadoxetic acid MRI for NET patients with suspected liver metastases being considered for surgery or liver-directed therapies, such as focal tumor ablation or arterial chemoembolization. This approach will leverage the high detection rate of NLM, including the identification of subcentimeter liver deposits on pMR, along with whole-body staging for extrahepatic metastases with DT-PET.

There are limitations to our study. First, it is retrospective and single-institution, including a relatively small patient cohort. Larger-scale prospective studies are needed to validate the performance of pMR and DT-PET (in sequence or using combined PET/MR) for the selection of patients and planning of liver-directed therapies. Second, the patient population was heterogeneous in terms of site of primary tumor, grade, and Ki67 index, and therefore, the concordance rate between the imaging modalities may not be applicable in cohorts with a higher proportion of higher-grade tumors, in whom pMR should be obtained when considering liver-directed therapies. Third, we allowed for a time interval of up to 4 months between pMR and DT-PET, and this may have skewed the data in favor of the study performed last. However, well-differentiated NETs are generally indolent, and our mean time interval between exams was less than 2 weeks, with 40% of patients receiving DT-PET following pMR and the rest prior to pMR, likely limiting the impact of this on the study results. Fourth, we limited documentation of deposits to up to five lesions per liver segment, and it is conceivable that in segments with more than five deposits, one of the modalities identified more lesions than the other without it being translated into the reported performance measures. However, in patients with extensive metastatic disease, the number of lesions within a segment beyond five lesions is unlikely to alter the therapy management plan. Finally, MR sequences were not assessed independently in a blinded fashion. Although this may introduce potential bias in terms of assessing the contribution of each sequence to the diagnostic performance of pMR, this does not impact the main objective, which is to determine the detection of pMR as an imaging modality.

## 5. Conclusions

In consecutive patients with well-differentiated NETs, the detection rate of NLM is higher with pMR than with DT-PET. However, when excluding patients whose tumors do not overexpress somatostatin receptors (13% of the cohort), high concordance in the detection of NLM is observed between DT PET and pMR. Further large-scale studies are needed to assess the role of ^68^Ga-DOTATATE PET/hepatobiliary-specific contrast-enhanced MR in the selection of patients with metastatic NET for liver-directed management and to determine its impact on patient outcomes. 

## Figures and Tables

**Table 1:** Cohort characteristics.

Variable	Category	Summary
Age at CT, Median (Min, Max)		57.5 (30.0, 80.0)
Gender, n (%)	Female	18 (60)
	Male	12 (40)
Primary site, n (%)	Small bowel	14 (47)
	Pancreas	10 (33)
	Lung	2 (7)
	Unknown	1 (3)
	Other	3 (10)
Tumor grade, n (%)	G1	12 (40)
	G2	12 (40)
	G3	6 (20)
Ki 67 index, n (%)	<3%	12 (40)
	3–20%	12 (40)
	>20%	6 (20)
Metastases, n (%)	Yes	23 (77)
	No	7 (23)

G1= grade 1; G2 = grade 2; G3 = grade 3.

**Table 2:** Lesion imaging characteristics on DT-PET. There were 75 segments with ≥1 metastasis on DT-PET.

Distribution of Modified Krenning Scores		
	Modified Krenning Score	*n* = (%)
Maximal per segment	4	53 (71)
	3	20 (27)
	2	2 (3)
	1	0 (0)
Minimal per segment	4	32 (43)
	3	41 (55)
	2	2 (3)
	1	0 (0)
**SUV metrics**		
Maximal SUVmax	Mean ± SD (range)	37.7 ± 26.7 (5.5, 146.6)
Minimal SUVmax	Mean ± SD (range)	19.7 ± 11.2 (4.4, 54.8)

SD = standard deviation.

**Table 3:** Lesion imaging characteristics on pMR. There were 98 segments with ≥1 metastasis on pMR.

Variable		*n* = (%)
T2 signal intensity	High	46 (47)
	Intermediate	45 (46)
	Low	7 (7)
Restricted Diffusion	Yes	85 (87)
	Equivocal	2 (2)
	No	11 (11)
Arterial Enhancement	Yes	72 (73)
	No	26 (27)
Wash Out	Yes	75 (77)
	No	23 (23)
Visible on HBP	Yes	98 (100)
	No	0

HBP = hepatobiliary phase.

**Table 4:** Liver metastasis count per patient.

Patient	pMR	DT-PET	Count Difference
1	0	0	0
2	11	11	0
3	11	1	10
4	4	5	−1
5	10	9	1
6	0	0	0
7	0	0	0
8	3	5	−2
9	3	0	3
10	44	42	2
11	45	36	9
12	0	0	0
13	19	0	19
14	0	0	0
15	11	11	0
16	2	3	−1
17	17	0	17
18	0	0	0
19	10	10	0
20	2	0	2
21	5	2	3
22	4	3	1
23	1	1	0
24	21	18	3
25	1	1	0
26	17	17	0
27	10	10	0
28	0	0	0
29	30	1	29
30	6	8	−2

Highlighted in grey are patients that were outliers, in whom significant disease was identified on pMR but no or small volume disease on DT-PET.

## Data Availability

The data presented in this study are available on request from the corresponding author.
